# The EQ-5D-5L and Minimal Important Change in Long COVID

**DOI:** 10.1177/27536351261423961

**Published:** 2026-02-21

**Authors:** Adam B. Smith, Darren C. Greenwood, Ruairidh Milne, Mike Ormerod, Manoj Sivan

**Affiliations:** 1 Academic Department of Rehabilitation Medicine, Leeds Institute of Rheumatic and Musculoskeletal Medicine, University of Leeds, UK; 2 Leeds Institute for Cardiovascular and Metabolic Medicine, School of Medicine, University of Leeds, UK; 3 Leeds Institute for Data Analytics, University of Leeds, UK; 4 Person with Long COVID, Long Covid Support, School of Healthcare Enterprise and Innovation, University of Southampton, UK; 5 Person with Long COVID, Long Covid Support, London, UK; 6 Covid Rehabilitation Service, Leeds Community Healthcare NHS Trust, UK; 7 National Demonstration Centre of Rehabilitation Medicine, Leeds Teaching Hospitals NHS Trust, UK

**Keywords:** long COVID, minimal important difference, minimal clinically important change, EQ-5D-5L

## Abstract

**Introduction:**

The EQ-5D-5L is the most commonly used patient-reported outcome measure in Long COVID (LC). Despite its frequent use, there have been few studies reporting LC-specific metrics to identify and interpret meaningful change. The aim of the study was therefore to determine the Minimal Clinically Important Difference (MCID) and Minimal Important Difference (MID) measures for the EQ-5D-5L in LC.

**Methods:**

Data were collected from a national study (LOCOMOTION) evaluating LC services in the UK, involving participants completing the EQ-5D-5L on at least 2 occasions. The EQ-5D domains were categorised using Paretian classification of health states, and the probability of superiority was used to determine changes in health states over time. EQ-5D-5L profile scores were converted into health utilities using the UK-specific algorithm. The MCID was derived using 0.5 standard deviation and the MID by a 0.2 effect size.

**Results:**

A total of 423 people (283 females, 67%) with LC completed the EQ-5D at 2 time points (median time interval: 196 days). Most participants reported problems in at least 1 EQ-5D domain. Only around 25% of participants noted some improvement. The MCID estimates were 0.11 for the EQ-5D-5L and 10.6 for the EQ-5D-5L VAS. The MID for the EQ-5D-5L was 0.03. Some differences in the change metrics were observed depending on baseline health states and timing of the follow-up assessment.

**Conclusion:**

Long COVID specific estimates of the MCIDs and MIDs were derived for the EQ-5D-5L and EQ-5D VAS. The MCIDs will facilitate the evaluation and interpretation of meaningful change in patient health states in LC, both at the individual level and more broadly in health economic assessments of LC management, intervention and rehabilitation programmes.

## Introduction

Post-COVID-19 Syndrome or Long COVID (LC) is a multi-organ syndrome defined as a persistence of symptoms around 3 months after a probable or confirmed SARS-CoV-2 infection and lasting for at least 2 months after the infection in the absence of alternative diagnoses.^1-3^ Global estimates suggest 10% to 20% of individuals may have developed LC following SARS-CoV-2 infection.^3,4^ These estimates are between 3% and 10% for the United Kingdom.^5,6^

Long COVID is characterised by over 200 symptoms, the most commonly reported being fatigue, pain, sleep problems, anxiety and depression.^7,8^ Symptoms fluctuate over time in response to physical, cognitive and emotional exertions.^7,9,10^ Assessment of health-related quality of life (HRQoL) – through the use of patient-reported outcome measures (PROMS) – is consequently important to evaluate the trajectory of patient symptoms, functioning, as well as to assist in the clinical management of LC. These aspects of LC may be captured with LC-specific PROMs,^11^ however, other measures, that is, preference-based measures (PBM) are required for the economic evaluation of LC interventions and healthcare service planning. These PBM instruments capture societal preferences for health states,^12^ in the form of health utilities, which may then be used, for instance, to inform healthcare resource allocation.^13^

The EuroQol 5-Dimension (EQ-5D)^14^ is one of the most frequently used PBM in LC^15^ and has been used in determining the impact of LC on work productivity,^16,17^ and population health-related quality of life following the pandemic,^18,19^ as well as in the evaluation of cost-effectiveness of rehabilitation programmes for LC.^20^

One important adjunct to aid the use of PROMs and PBMs is a metric for interpreting change, specifically meaningful change. Several definitions and methodologies have been proposed and employed.^21^ The common theme throughout these is a minimal change (as measured with the self-reported measure, such as a PROM or PBM) that is perceived as beneficial by the patient,^22^ that is a Minimal Important Difference (MID). The difference that are perceived as clinically important for individual patients and patient management are described as Minimal Clinically Important Differences (MCID).

Previous studies reporting MID metrics for the EQ-5D in LC have either used MIDs from medical conditions other than LC^23^ or have utilised MID based on population estimates.^24,25^ These MID estimates have ranged from 0.024 to 0.10. Few studies to date have determined MCIDs; one recent study^26^ that had highly functioning LC patients, proposed a seemingly high MCID value (0.262) for the EQ-5D-5L in LC.

Given the wide range of change metrics either used or estimated to date, the aim of the study was therefore to determine the MID and MCID for the EQ-5D-5L in LC with a wide range of functional disabilities.

## Methods

### Data

The data were derived from the LOng COvid Multidisciplinary consortium Optimising Treatments and servIces acrOss the NHS (LOCOMOTION) study.^27^ LOCOMOTION was a prospective mixed-methods study involving 10 LC services across the United Kingdom. Participants were eligible for inclusion if they had received a clinical diagnosis of LC by a qualified healthcare professional. Additionally, participants had to meet the UK National Institute for Health and Care Excellence (NICE) case definition, that is, 1 or more persistent symptoms developed during or post-infection that are consistent with COVID-19 and not explained by alternative diagnoses.^1^ Participants (with a diagnosis of LC) were recruited through the LC services where they were receiving assessments and management of LC. Patient-reported outcome measures were completed on a digital PROM platform developed by the digital health company ELAROS 24/7 Ltd and the University of Leeds.^28^ Ethics approval for the LOCOMOTION study was obtained from the Bradford and Leeds Research Ethics Committee on behalf of Health Research Authority and Health and Care Research Wales (reference: 21/YH/0276). No exclusion criteria were applied in the data extraction.

### Instruments

#### The EuroQol 5D-5L (EQ-5D-5L)

The EuroQol EQ-5D-5L has 5 dimensions^14^: Mobility, Usual Activities, Selfcare, Pain / Discomfort, and Anxiety / Depression. Each dimension has 5 response categories ranging from 1 (no problems) to 5 (severe problems). Responses to each dimension provide a profile score. This is converted into a health utility or index score using a country-specific algorithm (value set). Utilities are measured on a metric indexed at 0 (dead) to 1 (perfect health). Utility values less than 0, indicating states worse than dead, are also captured. The EQ-5D-5L also comprises a visual analogue scale (VAS) measuring self-reported current health on a scale from 0 (“worst health”) to 100 (“best health”).

The EQ-5D-5L scores were converted into EQ-5D-3L utilities using the crosswalk (CW) algorithm (mapping the 5-level EQ-5D onto the 3-L version) to derive UK utility values (EQ-5D-5L)^29^ currently recommended by the UK’s National Institute for Health and Care Excellence (NICE).^13^

#### Analysis

The Paretian Classification of Health Change PCHC)^30^ was used to categorise individual health changes into “better” (improvement on ≥1 dimensions), “worse,” (deterioration 1 ≥ dimensions), “mixed” (both improvement and deterioration in dimensions) and no change. Changes in individual health states were further categorised using the non-parametric effect size measure, probability of superiority (PS).^31^ For each EQ-5D dimension, the number of individuals with improvement at follow-up was divided by the total number of matched pairs. This metric is >0.5, if more participants improve; <0.5 if more deteriorate and 0.5 if the number improving equals the number of participants deteriorating.

The mean EQ-5D Index scores were derived for both the EQ-5D-5L and VAS at baseline (first visit) and follow-up, as well as the change from baseline scores. The time to follow-up represents the maximum time, that is, the most recent time to completion of the EQ-5D.

Two main methodologies exist for the estimation of meaningful change of patient-reported outcome measures, that is, anchor- and distribution-based approaches. The former relies on an external criterion – the anchor – such as a patient’s evaluation of change in their health status against which to determine the MID/MCID. Distribution-based approaches rely on sample parameters, rather than external criteria, to estimate the MID/MCID. Commonly used metrics include the effect size, standard of error measurement (SEM), and the standard deviation (SD).^21^ The latter (SD), in the form of half a standard deviation, is perhaps the most frequently cited metric as a robust estimate of the MCID.^32^ In the absence of participants’ self-reported change in health state, the 0.5 standard deviation (SD; change from baseline) metric was therefore used to estimate the MCID. Further MCID estimates were also derived for the data split by median baseline EQ-5D scores, as well as median time to follow-up to determine whether the MCID is dependent on baseline health status and whether the level of the metric changes over time. The 0.2*effect size (0.2ES) was derived as an estimate of the MID (for the EQ-5D-5L alone), the smallest change that could, for instance be employed to evaluate differences between groups of patients (for instance, in a clinical trial or population health evaluations). The 0.2ES was calculated using the change from baseline divided by the baseline SD. The analysis was undertaken in R (version 4.4.2).

## Results

Data were only extracted for those participants with at least 2 completed assessments including the EQ-5D. A total of 423 participants completed the EQ-5D at the 2 time intervals (Table 1); 67% (N = 283) were female, with an average age of 46 years. The sample was predominantly White (89%, N = 375). Close to two-thirds (64%, N = 272) of the sample had no pre-COVID co-morbidities; 24% (103) had 1 and 11% (48) had 2 or more co-morbidities. The mean time to completion of the second EQ-5D (follow-up) was 208 days (median: 196 days; maximum: 659 days; inter-quartile range (25%-75%): 23-251 days).

**Table 1.** Sample Demographics.

Demographics	Female	Male
N = 423 (%)	283 (67)	140 (33)
Age (Mean, standard deviation)	45.8 (12.7)	46.3 (12.5)
Ethnicity (N, %)		
White (includes any White background)	252 (89.1)	123 (87.9)
Asian (includes any Asian background, for example, Bangladeshi, Chinese, Indian, Pakistani)	11 (3.9)	6 (4.3)
Black, African, Black British or Caribbean (includes any Black background)	7 (2.5)	4 (2.9)
Mixed or multiple ethnic groups (includes any Mixed background)	7 (2.5)	2 (1.4)
Another ethnic group (includes any other ethnic group, for example, Arab)	2 (0.7)	3 (2.1)
Not reported	4 (1.4)	2 (1.4)

Abbreviations: N, number; LC, long COVID.

### EQ-5D-5L Profiles

A significant number of participants reported problems across the 5 EQ-5D domains (Table 2). This was particularly evident for “usual activities” (96.5%), as well as “pain/distress” (91.3%) and “anxiety/depression” (91%). Similarly, 52% reported no problems with “self-care,” only 24% with “mobility” and less than around 10% were experiencing no problems with the other 3 domains. This was reflected in the fact that only around a third of participants recorded some improvement across the EQ-5D domains. Furthermore, the probability of success only indicated a marginal improvement in participants’ health status.

**Table 2.** Health State Change.

Paretian classification of health change/EQ-5D domains	Mobility	Self-care	Usual activities	Pain/distress	Anxiety/depression
No change	140 (43.2)	66 (32.7)	157 (38.5)	184 (47.7)	162 (42.1)
Improved	106 (32.9)	64 (31.7)	167 (40.9)	122 (31.6)	137 (35.6)
Worsened	77 (23.9)	72 (35.6)	84 (20.6)	80 (20.7)	86 (22.3)
Total with problems	322 (76.1)	202 (47.8)	408 (96.5)	386 (91.3)	385 (91.0)
No problems	101 (23.9)	221 (52.2)	15 (3.5)	37 (8.7)	38 (9.0)
Probability of superiority (PS)	0.53	0.49	0.6	0.55	0.56

### EQ-5D Minimal Clinically Important Difference

The results for the EQ-5D Index scores and MCIDs are shown in Table 3. The mean baseline score for the EQ-5D-5L was 0.52 (SD: 0.25; median 0.555; minimum: −0.346; 0.358-0.7 (25th-75th centile); for the VAS this was 48.0 (SD: 20.5; median: 50.0; minimum: 0; 30.0 to 64.0 (25th-75th centile).

**Table 3.** EQ-5D-3L Index and VAS Scores and Minimal Clinically Important Differences.

EQ-5D / time	Baseline	Follow-up	Change	MCID	MID
OverallEQ-5D-5L	0.52 (0.25)	0.558 (0.263)	0.039 (0.211)	0.1055	0.031
EQ-5D-3L (⩽ 0.358; N = 106)^a^	0.166 (0.145)	0.313 (0.26)	0.146 (0.241)	0.1205	0.201
EQ-5D-3L (>0.358; N = 317)	0.638 (0.144)	0.64 (0.207)	0.003 (0.186)	0.093	0.004
EQ-5D-3L (⩽196 d; N = 212)	0.521 (0.247)	0.553 (0.251)	0.032 (0.182)	0.091	0.026
EQ-5D-3L (>196 d; N = 211)	0.518 (0.254)	0.564 (0.274)	0.046 (0.236)	0.118	0.036
Overall EQ-5D VAS	48.01 (20.54)	53.23 (21.17)	5.22 (21.14)	10.6	-
EQ-5D VAS (⩽ 0.358; N = 106)	33.04 (18.2)	39.95 (20.07)	6.92 (22.35)	11.2	-
EQ-5D VAS (>0.358; N = 317)	53.02 (18.78)	57.67 (19.64)	4.65 (20.72)	10.4	-
EQ-5D VAS (⩽196 d; N = 212)	49.9 (20.52)	51.34 (20.48)	1.43 (19.56)	9.7	-
EQ-5D VAS (>196 d; N = 211)	46.11 (20.43)	55.13 (21.72)	9.01 (22.01)	11.0	-

Abbreviations: AS, visual analogue; MCID, minimal clinically important change; MID, minimal important difference.

a EQ-5D-3L value set.

Only a small change from baseline was observed for both EQ-5D-5L (0.04) and VAS (5.2) reflecting the lack of improvement observed in the profiles.

The overall MCID estimates were 0.106 and 10.6, respectively, for the EQ-5D-5L and VAS. The overall mean change for both versions did not exceed the individual MCIDs, suggesting that as a group there was no minimally clinically significant change over time. Only 32% of the sample exceeded the MCID for the EQ-5D-5L (N = 136) and 35% for the VAS (N = 156).

There was a baseline effect observed with the MCID higher (0.121, EQ-5D-5L) for those participants with lower health utilities at baseline compared to those with higher baseline scores (0.093). This was observed to a lesser extent for the VAS (11.2 and 10.4 respectively). The baseline score difference for MCID was also associated with a greater change from baseline, for example, 0.146 for those with lower baselines and 0.003 for those with higher baselines (for the EQ-5D-5L). Again, this was also observed for the VAS but to a smaller degree.

In addition to this, slightly lower MCIDs were demonstrated for follow-up visits within (eg, 0.091) compared to after the median 196 days (0.118) for the EQ-5D-5L. This was also the case for the EQ-5D 5L VAS. As with the baseline split, there were also differences in the degree of change: shorter periods were associated with smaller changes from baseline compared to longer follow-up periods.

The minimal important difference (MIDs) was 0.031 for the EQ-5D-5L. Almost half (49%.2) of the sample achieved a change score equivalent to or greater than this MID. As with the MCID, differences were observed by baseline score and duration to follow-up. However, the MID for those participants with baseline EQ-5D-5L scores below the 25th centile (0.358) was significantly higher (0.201), suggesting a greater degree of change would be required for patients to observe a minimal change in their health status.

## Discussion

The aim of the study was to derive estimates for the minimal (clinically) important differences (MCID/MID) for the EQ-5D in LC. The MID for EQ-5D-5L index value was estimated to be 0.031 and for EQ-5D-5L VAS was 4.2. The MCID estimates were 0.11 for the EQ-5D-5L and 10.6 for the EQ-5D-5L VAS. It should be noted that little improvement over time was noted in health states with the majority of participants reporting problems with at least one dimension of the EQ-5D.

The MID estimates fell within the range of previously published studies based either on population estimates or derived from other medical conditions.^23-25^ The MID of 0.031 (for the EQ-5D-5L) may be suitable for population-based evaluations or assessing group differences in clinical trials or interventions. However, it should be noted that the MID, in particular, was dependent on baseline EQ-5D-5L scores, with the results indicating that a significantly larger MID might be warranted for those populations with low health-related quality of life (EQ-5D-5L <0.358). For instance, in line with the literature on the EQ-5D in general,^33^ the results demonstrated some baseline effects: higher MCIDs (both versions) were shown for participants with baseline scores below the 25th centile, indicating that a greater degree of improvement in health states is required for these participants in terms of meaningful change. This was also reflected in the degree of change observed. At the same time, those participants with higher baseline scores had a lower MCID estimate by comparison, suggesting a smaller change required to be interpreted as a meaningful improvement.

Regarding the timing of the follow-up alongside change in EQ-5D index scores, shorter terms (within approximately 3-4 months of baseline) were associated with lower MCIDs than longer follow-up periods. The differences from baseline mirrored this and potentially indicate a slow initial improvement in participants’ health states during the earlier course of rehabilitation followed by a more rapid, albeit small improvement at a later stage. A smaller MCID (0.08) is therefore required in terms of interpreting change in the early stages of an intervention.

One study in the literature has reported large MCIDs (0.262) for the EQ-5D-5L in LC^26^ using a receiving operating characteristic (ROC) analysis. Although, as noted by those authors, the area-under-the-curve (AUC) in the analysis fell below the lowest acceptable limit for diagnostic thresholds (<0.70).^34^ However, when applying the 0.5SD metric to those data (Table 1, page 4), based on change from baseline, the derived MCIDs would fall between 0.08 and 0.084, agreeing more closely with the MCID for the EQ-5D-5L determined in the current study.

## Limitations

Anchor-based approaches are generally considered to be more robust methods for determining minimal important change.^21,35^ A corollary to this is that as LC is a highly fluctuating condition, the within-person standard deviation (SD for change) is potentially larger than for other more stable chronic conditions. Therefore, the MCID based on the SD for change will inevitably be quite large. This does not imply that people living with LC would not notice an improvement or worsening of their condition measurably less than the MCID. Instead, the research evidence and the lived experience suggests that they do indeed recognise these fluctuations.^9,36^ However, these changes need to be captured through self-report, which was absent in this study. The main limitation in this study was, therefore, the lack of external anchors, such as the global impression of change to provide a self-reported evaluation of change. In addition to this, the sample was selectively drawn from people attending LC services. Although the LC services were characterised by the multidisciplinary assessment and rehabilitation of patients with LC, these were delivered through diverse service models and staff mix,^37^ which may have impacted the change metrics. Heterogeneity of treatment modalities and LC services could possibly also impact on comparisons with other countries, however, as noted above, the MID values in this study fell within the range of previously published estimates. This suggests that the EQ-5D MID determined in the UK context is broadly comparable with those found across other healthcare systems.^24-26^

An additional potential limitation in terms of representativeness is the sample characteristics. The sample was reasonably representative of the LC population in the UK in terms of having a greater representation (67%) of female participants, as well as the mean age (approximately 46 years) falling within the published range (35-69 years).^6^ However, ethnic minorities were underrepresented in this sample, that is, 11% relative to the overall UK population^38^ of around 18%. Nevertheless, it should be noted that the sample was more representative of people with LC who have had the condition for longer duration, which also helps to explain the limited improvements observed.

Therefore, taken collectively, further research is required using anchor-based approaches to confirm the EQ-5D 5L MID/MCID estimates determined in this study in LC cases managed elsewhere (ie, not referred to LC services) as well as amongst minority ethnic groups.

In terms of choice of the value sets for the health utility indices, the EQ-5D-3L is currently the UK National Institute for Health and Care Excellence’s (NICE) preferred method for providing health utility values. Moreover, the organisation does not currently recommend the use of the -5L value set for health technology appraisals owing to quality concerns.^39^ This was the primary justification for using the -3L, rather than the -5L value sets. Although, this could potentially have impacted on sensitivity, further post hoc analysis applying the -5L value set to the data demonstrated an MCID of 0.095 and MID of 0.032. These values are close to those derived for the -3L (0.106 and 0.031 reported in Table 3) to suggest there was no detrimental effect on sensitivity.

Finally, there is also some evidence that the EQ-5D is not sufficiently sensitive to detect change in LC,^40^ therefore additional research is required to investigate MID/MCID changes in this instrument when compared to more sensitive LC measures such as the modified Covid-19-Yorkshire Rehabilitation Scale (C19-YRSm).^11^

## Conclusions

Long COVID-specific estimates of the MCID were derived for the EQ-5D-5L Index Value and EQ-5D VAS. The MCIDs will contribute to aiding the evaluation and interpretation of meaningful change in patient health states in LC both at the individual level, and societal leave. The MCID estimation will also be useful in health economic assessments of LC management, intervention and rehabilitation programmes.
